# Evaluating the Benefit of a Urogynecologic Telehealth Consultation after Obstetric Anal Sphincter Injury

**DOI:** 10.1007/s00192-025-06077-2

**Published:** 2025-01-31

**Authors:** Kyra Bonasia, Susan Luong, Jocelyn Stairs, Aisling Clancy

**Affiliations:** 1 Department of Obstetrics and Gynecology, University of Ottawa, Ottawa, Canada; 2 Department of Obstetrics and Gynecology, Dalhousie University, Halifax, Canada

**Keywords:** Obstetric anal sphincter injuries, Pelvic floor disorders, Peripartum perineal laceration, Postpartum health, Telehealth

## Abstract

**Introduction:**

Obstetric anal sphincter injuries (OASI) are associated with significant risk of complications, including pain, infection, and long-term pelvic floor dysfunction. The primary aim of this study was to evaluate the utility and acceptability of a postpartum telehealth consultation focused on pelvic floor health for patients after OASI.

**Methods:**

This prospective study used a pre–post design comparing standard postpartum care versus standard postpartum care plus a telehealth urogynecology consultation focused on pelvic floor recovery. The primary outcome was symptom burden as measured by the Pelvic Floor Distress Inventory (PFDI-20) score 16-weeks postpartum. Patient experience was evaluated using the QQ10 and the Patient Enablement Instrument. *T*-tests and chi-squared tests were used to compare groups.

**Results:**

A total of 119 participants completed study activities (control group *n* = 62, intervention group *n* = 57). There was no significant difference between the two groups in PFDI-20 scores (55.6 versus 46.6, *p* = 0.23). The individual items most likely to be endorsed among all participants were related to flatal incontinence (52.1%) and fecal urgency (49.6%). For the subset analysis of 35 patients with severe OASI (3C or fourth-degree tears), those who had a telehealth consultation had lower PFDI-20 scores (56.6 versus 34.7; *p* = 0.04). QQ10 estimated a value score of 79/100 and a burden score of 18/100 for the telehealth consultation.

**Conclusions:**

A postpartum telehealth consultation focused on pelvic floor health may benefit patients with severe OASI who reported reduced symptom burden. Participants rated a telehealth consultation as high value and low burden for this condition.

**Supplementary Information:**

The online version contains supplementary material available at 10.1007/s00192-025-06077-2.

## Introduction

At the time of vaginal delivery, approximately 5% of people sustain third- or fourth-degree perineal lacerations known as obstetric anal sphincter injuries (OASI) [1]. OASIs increase risk of complications during recovery, including pain and risk of postpartum wound complications [2, 3]. OASIs are also associated with long-term pelvic floor dysfunction years after delivery, including sexual dysfunction, anal incontinence (AI), and urinary incontinence (UI) [4, 5]. These symptoms are not rare, with half of women reporting at least one symptom occurring “frequently” four years after OASI [4], and the mean rate of AI following primary repair of OASI is estimated at 39% [6]. Even those without persistent symptoms after OASI require careful evaluation and counselling due to the risk of new or worsening symptoms associated with aging or after a subsequent delivery [7, 8]. For example, those with abnormalities on assessment of the anal sphincter after OASI have an increased risk of long-term AI if they have another vaginal delivery [1, 9].

Routine postpartum care in North America involves a single visit approximately 6 weeks after delivery addressing physical recovery, breastfeeding, contraception, and mood. Although dedicated postpartum perineal follow-up is not standard in North America, postpartum perineal clinics have been described in the literature [10, 11]. They can provide dedicated streamlined counselling, planning for future deliveries, acute care for issues such as wound complications, referral for investigations such as anal manometry and ultrasound, and may facilitate further care when needed.

Accessing a dedicated perineal clinic may be challenging for patients living in areas remote from specialist care and patients caring for a newborn. Moreover, many patients affected by incontinence do not seek assistance from a health care provider, often due to social stigma and embarrassment [12, 13]. Telehealth postpartum perineal follow-up may provide a more accessible way for patients to seek care. Telehealth visits for general postpartum care became more widely offered during the COVID-19 pandemic, with patients reporting high satisfaction and benefits related to comfort, convenience, communication, and socioeconomic factors [14]. Virtual postpartum interventions have shown benefit for postpartum depression [15], weight loss [16], and lactation support [17]. There are, however, no reports of postpartum telehealth interventions focused on pelvic floor health.

The primary aim of this study was to evaluate the utility and acceptability of a telehealth consultation with a urogynecologist at 8–12 weeks postpartum after OASI. Secondary aims were to evaluate the need for additional postpartum follow-up focused on pelvic floor health as measured by the proportion of patients requiring further interventions or referrals, and to estimate the prevalence of pelvic floor symptoms at 16-weeks postpartum after OASI.

## Materials and Methods

### Study Design

This is a prospective cohort study, with a pre–post design. The control group was recruited first (August 2022 to June 2023) and received standard postpartum care. The intervention group was recruited second (July 2023 to February 2024) and received standard postpartum care plus an additional telehealth consultation with a urogynecologist at 8–12 weeks postpartum. The pre–post design was chosen to prevent cross-over or contamination between participant groups as there is significant communication between patients in the obstetric community in our region (particularly through social media groups for new parents and/or for people who have experienced certain peripartum complications). Telehealth visits are not standard practice for postpartum care in our region.

### Study Population

Participants in the study met the following inclusion criteria: 18 years or older, English or French speaking, vaginal delivery, singleton birth, delivery at 36 weeks gestational age or later, third- or fourth-degree perineal laceration associated with delivery. Participants were recruited via referral from a member of the patient’s healthcare team. Deliveries occurred at our tertiary care centre with approximately 6800 deliveries per year by obstetricians, family medicine physicians and midwives at two hospital sites. Standard practice is for patients delivering under midwifery or family medicine physician care to be referred to an obstetrician for immediate repair of severe perineal lacerations (including OASI).

### Intervention

Participants in the control group received standard postpartum care, with the scheduling and number of visits determined by each patient’s care provider (obstetrician, family physician, or midwife). For most patients this would be a follow-up visit approximately 6-weeks postpartum, with further investigations, interventions, or referrals made at the discretion of their care provider.

Participants in the intervention group received standard postpartum care, as well as an additional urogynecology telehealth phone consultation at 8–12 weeks postpartum. This telehealth consultation focused on pelvic floor symptoms and resumption of routine activities, addressing pelvic health concerns, and (if relevant) discussing future pregnancy planning. These telehealth consultations were conducted by either a urogynecologist or urogynecology fellow. The timing of this visit was chosen to occur after the standard 6-week postpartum follow-up, to assess the need for additional resources or follow-up after completion of standard postpartum care. Participants were referred for in-person assessment, investigations, and/or treatment as needed.

### Data Collection

Demographic data was collected during referral from a member of the participant’s healthcare team. Participants in both cohorts completed an anonymous online survey at 16-weeks postpartum, via a link sent to their email address, which included the validated questionnaires listed below. See Supplemental Information 1 for the survey questionnaires. Participants in the telehealth cohort had data regarding their consultation recorded by the telehealth provider.

### Outcome Measures

The primary outcome was Pelvic Floor Distress Inventory (PFDI-20) cumulative score at 16-weeks postpartum [18]. Secondary outcomes included the following: individual scores for the three domains within the PFDI-20—Pelvic Organ Prolapse Distress Inventory (POPDI-6), Colorectal-Anal Distress Inventory (CRADI-8), and Urinary Distress Inventory (UDI-6); Patient Enablement Instrument (PEI) a validated questionnaire assessing a patient’s ability to understand and cope with illness and life after a medical consultation [19, 20]; QQ10 a questionnaire developed to measure the value and burden of patient experience [21], which has been modified and used to evaluate patient experience of telehealth consultations [22]; and the proportion of patients requiring additional follow-up, including investigations, interventions, or referrals.

We collected information on relevant covariates, including age, maternal pre-pregnancy body mass index (BMI), birthweight of baby, type of vaginal birth (spontaneous, vacuum-assisted, forceps-assisted), number of vaginal deliveries, number of caesarean deliveries, pelvic health concerns prior to most recent delivery, and presence of private health insurance. Perineal lacerations were classified as either partial third-degree (3A or 3B), complete third-degree (3C), or fourth-degree.

### Statistical Analyses

To detect a 10-point reduction in PFDI-20 score (previously determined to be clinically significant) [23], assuming an average baseline score of 40 out of 100 and an estimated standard deviation of 20 (based on previous data [24]), we aimed to recruit a sample size of 128 participates (64 per group), providing a power of 0.8.

We performed *t*-tests for continuous variables and chi-squared tests for categorical variables to evaluate for differences in baseline patient characteristics between control and intervention groups and to evaluate study outcomes. A subset analysis was completed for patients with severe OASI (3C or fourth-degree tears) given the known higher rates of pelvic floor dysfunction among these patients. As part of this subset analysis, a multivariable linear regression model was completed to evaluate for differences in PFDI-20 score between the cohorts, adjusting for age, BMI, and number of vaginal births.

The POPDI-6, CRADI-8, and UDI-6 scores were calculated by obtaining the mean value of all the items within the corresponding scale and multiplying by 25 (range 0 to 100). The PFDI-20 summary score was calculated by adding the three component scales together (range 0 to 300). Missing data in the PFDI-20 (0.2% of responses missing) were replaced with the mean of the responses in the same category, per PFDI-20 described scoring protocol. PEI scores were calculated by assigning a score of 0–2 for each of the six items (total score range 0 to 12), with higher scores representing greater patient enablement. For the PEI, one participant in the control group did not answer this section of the survey, only complete responses were used for comparison. The QQ10 value and burden scores were calculated by adding the scores of the six “value” items and the four “burden” items, followed by transformation of the scores onto a scale of 0–100, with 100 representing the best possible value score, and 0 representing the best possible burden score [21, 22].

Data analyses were completed using STATA, version 15.1.

## Results

**Fig. 1:**
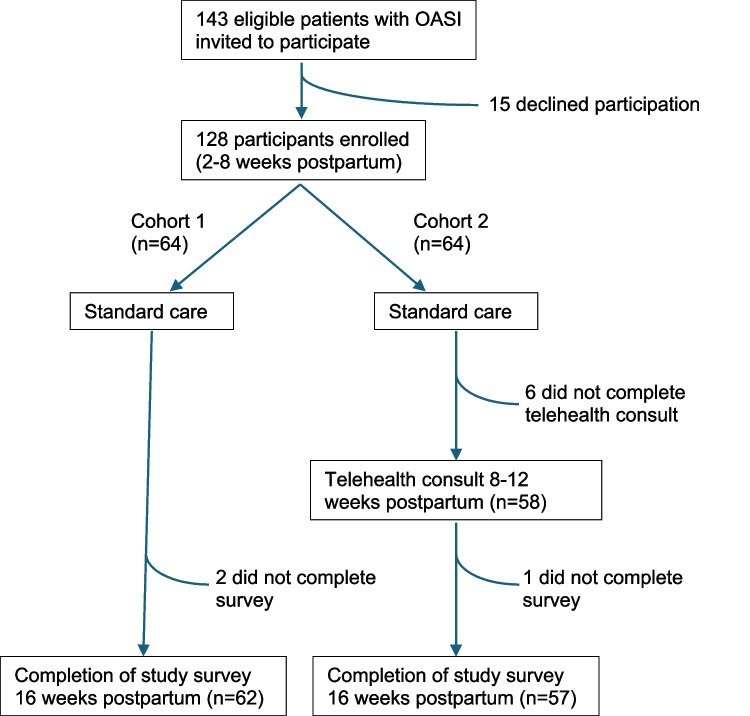
Flow-chart outlining study recruitment, intervention, and data collection. Abbreviations: obstetric anal sphincter injury (OASI)

A total of 128 participants met inclusion criteria and were enrolled in the study, 64 in each group, out of 143 eligible patients who were contacted for possible participation (89.5% recruitment rate). In the control group, 62 participants completed the study activities (retention rate 97%), in the intervention group, 57 participants completed the study activities (retention rate 89%) (Fig. 1). There were no significant differences between the control and intervention groups with respect to baseline characteristics. Mean age of each group was 32.3 years (SD 3.8) and 31.1 years (SD 3.9), respectively (*p* = 0.09), while mean pre-pregnancy BMI was 27.2 (SD 6.8) and 26.5 (SD 5.2) (*p* = 0.50). For most patients, this was their first vaginal delivery (89% and 93%, respectively, *p* = 0.42), with no significant difference in the rates of spontaneous versus assisted delivery (vacuum and forceps assisted deliveries). Mean baby birthweights were 3453 g (SD 461 g) and 3390 g (SD 421 g), respectively (*p* = 0.42). The majority of participants experienced partial third-degree tears (66% and 75%, respectively, *p* = 0.51). Most participants had no pre-existing pelvic floor concerns (90% and 86%, respectively, *p* = 0.68), with urinary incontinence identified as the most common pre-existing pelvic floor concern (10% and 12% of participants in each group). Baseline characteristics are reported in Table 1.

**Table 1:** Baseline characteristics for the two study groups

Characteristic	Control group *n* = 62	Intervention group *n* = 57	*P* value
Mean age, years (SD)	32.3 (3.8)	31.1 (3.9)	0.09
Mean pre-pregnancy BMI, kg/m2 (SD)	27.2 (6.8)	26.5 (5.2)	0.50
Mean baby birthweight, g (SD)	3453 (461)	3390 (421)	0.42
First vaginal delivery, *n* (%)	55 (89%)	53 (93%)	0.42
Type of vaginal delivery, *n* (%)			
• Spontaneous	33 (52%)	30 (53%)	0.31
• Vacuum assisted	25 (42%)	20 35%)	
• Forceps assisted	3 (5%)	7 (12%)	
Type of perineal tear			
• Partial third-degree (3A or 3B)	41 (66%)	43 (75%)	0.51
• Complete third-degree (3C)	16 (26%)	10 (18%)	
• Fourth-degree	5 (8%)	4 (7%)	
Pre-existing pelvic floor concerns, *n* (%)^a^			
• Urinary incontinence	6 (10%)	7 (12%)	0.68
• Flatal incontinence	1 (2%)	3 (5%)	
• Fecal incontinence	0	0	
• Pelvic organ prolapse	0	0	
• None	56 (90%)	49 (86%)	
Private health insurance in addition to provincial health insurance, *n* (%)	46 (74%)	48 (84%)	0.18

The control group received standard postpartum care. The intervention group received standard postpartum care plus an additional telehealth phone consultation with a urogynecologist at 8–12 weeks postpartum

*SD* standard deviation, *BMI* body mass index

^a^Note that this sums to > 100% due to 1 participant in the control group and 2 participants in the intervention group endorsing both urinary and flatal incontinence

For the pelvic floor symptom analysis (Table 2), there was no significant difference between the two groups in PFDI-20 scores, control group 55.6 (SD 42.9) versus intervention group 46.6 (SD 37.5) (*p* = 0.23), or in any of the subsection scores. The individual items on the PFDI-20 that were most likely to be endorsed were in the Colorectal-Anal Distress Inventory (Supplemental Table 1 for summary of individual item results). The two statements endorsed most frequently among all participants were “Do you usually lose gas from the rectum beyond your control?” (endorsed by *n* = 62, 52.1%) and “Do you experience a strong sense of urgency and have to rush to the bathroom to have a bowel movement?” (endorsed by *n* = 59, 49.6%).

**Table 2:** Questionnaire scores for the Pelvic Floor Distress Inventory (PFDI-20), including its three sub-sections: Pelvic Organ Prolapse Distress Inventory (POPDI-6), Colorectal-Anal Distress Inventory (CRADI-8), and Urinary Distress Inventory (UDI-6); and for the Patient Enablement Instrument (PEI)

Questionnaire	Control group *n* = 62	Intervention group *n* = 57	*P* value
PFDI-20 mean score (SD)^a^	55.6 (42.9)	46.6 (37.5)	0.23
• POPDI-6	14.2 (14.2)	10.8 (12.4)	0.17
• CRADI-8	19.3 (16.5)	18.6 (19.0)	0.85
• UDI-6	22.1 (22.6)	17.1 (16.5)	0.17
PEI mean score (SD)^b^	5.2 (4.1)	5.5 (3.3)	0.72

Mean scores are provided with standard deviations (SD)

^a^PFDI-20 is out of a total of 300 and is calculated as the sum of the three sub-section scores. Each sub-section score is out of a total of 100

^b^PEI is out of a maximum score of 12

For the subset analysis of patients with severe OASI (3C or fourth-degree tears), those in the intervention group had lower PFDI-20 scores (56.6 SD 33.5 versus 34.7 SD 22.5, *p* = 0.04) (Table 3). Lower scores were reported across all three subsections (POPDI-6, CRADI-8, UDI-6). A multivariable linear regression was performed, confirming that this result remained statistically significant (*p* = 0.04, R^2^ = 0.18, beta coefficient = −22.9) after adjusting for key confounders (age, BMI, number of vaginal deliveries).

**Table 3:** Subset analysis for the patients who sustained severe obstetric anal sphincter injuries, defined as complete third-degree (3C) or fourth-degree tears, *n* = 35

	Control group *n* = 21	Intervention group *n* = 14	*P* value
Demographics			
Mean age, years (SD)	31.0 (3.8)	29.8 (3.5)	0.20
Mean pre-pregnancy BMI, kg/m^2^ (SD)	27.2 (5.3)	27.6 (5.9)	0.86
Mean baby birthweight, g (SD)	3406 (440)	3534 (539)	0.45
First vaginal delivery, *n* (%)	20 (95%)	14 (100%)	0.69
Type of delivery, *n* (%)			0.47
Spontaneous vaginal	11 (52%)	5 (36%)	
Vacuum assisted	9 (43%)	5 (36%)	
Forceps assisted	1 (5%)	2 (14%)	
Questionnaire Outcomes			
PFDI-20 mean score (SD)	**56.6 (33.5)**	**34.7 (22.5)**	0.04*
• POPDI-6	13.3 (13.7)	9.2 (12.8)	0.38
• CRADI-8	17.1 (12.2)	12.1 (7.1)	0.18
• UDI-6	26.3 (21.8)	13.4 (13.2)	0.06

*SD* standard deviation, *BMI* body mass index, *PFDI-20* Pelvic Floor Distress Inventory, *POPDI-6* Pelvic Organ Prolapse Distress Inventory, *CRADI-8* Colorectal-Anal Distress Inventory, *UDI-6* Urinary Distress Inventory

^*^p < 0.05

There was no significant difference between groups on the PEI, control group 5.2 (SD 4.1) versus intervention group 5.5 (SD 3.3) (*p* = 0.72) (Supplemental Table 2 for summary of individual PEI items). QQ10 was completed by the intervention group to evaluate the telehealth consultation, with a mean “value” score of 79 (SD 15), and a mean “burden” score of 18 (SD 19), both on a scale of 0–100; 73.7% agreed that they would be happy to have a telephone consultation again in the future as part of their routine care (Supplemental Table 3 for summary of individual QQ10 items).

During the telehealth consultations, which occurred at 8–12 weeks postpartum, the most commonly reported symptom was pain (50.9%, *n* = 29), followed by flatal incontinence (36.8%, *n* = 21), urinary incontinence (26.3%, *n* = 15), and fecal incontinence (17.5%, *n* = 10). Half of participants reported engagement with pelvic floor physiotherapy as part of their postpartum care (52.6%, *n* = 30). Almost half of participants (42.1%, *n* = 24) had additional follow-up recommended during their telehealth consultation, most commonly referred to pelvic floor physiotherapy (*n* = 19), with 3 participants referred to urogynecology, 1 participant referred to general gynecology, and 1 participant referred for endoanal ultrasound. A prescription for vaginal estrogen was provided to 40.4% of participants (*n* = 23). The use of vaginal lubricants and stool softeners or fibre and bulking agents as needed was discussed with all patients as part of the telehealth consultation. Telehealth consultations lasted an average of 14 minutes with a range of 10 to 20 minutes. See Supplemental Table 4 for summary of telehealth visit data.

## Discussion

This prospective study compared patients with OASI who received standard postpartum care versus standard postpartum care with an additional telehealth consultation focused on pelvic floor health. Although there were no significant differences on the PFDI-20 at 16-weeks postpartum when comparing all participants with OASI, when we restricted the analysis to those with severe OASI a telehealth consultation was associated with significantly lower PFDI-20 scores, suggesting some benefit in this subset of patients. Patients in both groups reported ongoing symptoms at 16-weeks postpartum, and the most commonly endorsed individual items on the PFDI-20 were related to flatal incontinence and fecal urgency. Almost half of patients had additional follow-up recommended during the telehealth consultation suggesting that standard postpartum care had not fully addressed pelvic floor concerns.

Telehealth interventions have been applied in postpartum populations for postpartum depression, lactation support, and weight loss [15–17], and telehealth care has been shown to be acceptable to patients post-operatively after urogynecologic surgery [25]. This study, however, is the first to evaluate the benefit of telehealth for postpartum care after OASI. The QQ10 has been used to evaluate patient experience of telehealth consultations for general urogynecology care, with that study reporting a mean value score of 77 and a mean burden score of 17 [22]. This is comparable to our findings for telehealth consultation after OASI (QQ10 mean value score of 79 and mean burden score of 18), with the majority of patients stating that they would be happy to have a telephone consultation again in the future.

Although we did not find a significant difference in PFDI-20 between the two groups, we suspect that this was due to higher-than-expected variability in PFDI-20 scores. For our power calculation we used an estimate of a 10-point reduction in PFDI-20, based on prior literature suggesting that difference to be clinically significant. We found a 9-point reduction; however, this was not significant in our statistical analyses, likely because the standard deviation in our PFDI-20 scores was approximately double the estimate in our power calculation (estimated SD 20, actual SD 43 and 38), suggesting high variability in symptom severity reported by participants. When we restricted the analysis to only those with severe OASI (3C and fourth-degree tears), we found significantly lower PFDI-20 scores in the telehealth consultation group compared to those who received standard care. Focusing postpartum pelvic floor resource development towards patients with severe OASI may have the greatest impact on pelvic floor health outcomes.

It is also possible that the timeframe between the telehealth consultation (8–12 weeks postpartum) and the assessment of symptoms (16 weeks postpartum) was too short to fully evaluate the effect of the intervention. For example, pelvic floor physiotherapy is recommended for all patients after OASI and was the most recommended intervention in the telehealth visits, due to only half of patients being engaged in pelvic floor physiotherapy after standard postpartum care. However, this is not expected to demonstrate a significant effect after only a month, and the other referrals (urogynecology, gynecology, endoanal ultrasound) would likely not have taken place in that time. Other recommended interventions, however, such as vaginal estrogen, lubricants, bulk-forming laxatives, and at-home exercises may have had an effect within this timeframe.

Most patients (82.5%) reported breastfeeding at the time of the telehealth consultation. The hypoestrogenic state associated with lactation has been reported to manifest as vaginal dryness, dyspareunia, and urinary symptoms [26], which in this study may have exacerbated the general perineal discomfort associated with OASI. While vaginal estrogen has been proposed as an intervention for the genitourinary symptoms associated with lactation [26] and was prescribed to many patients during the telehealth visits, there are limited studies assessing this. One pilot randomized controlled trial showed minimal clinical benefit; however, the study size was small, and enrollment was stopped early due to the COVID-19 pandemic [27]. Future research could assess the benefits of vaginal estrogen in postpartum lactating patients, particularly in those with OASI who are at higher risk of postpartum pain and dyspareunia.

For the Patient Enablement Instrument (PEI), a commonly used cut-off is a score of 6-points as reflecting “high” enablement [28]. This study revealed relatively low PEI scores for both groups (5.2 and 5.5, respectively), suggesting low enablement in this population of postpartum patients after OASI. This is consistent with a recent literature review reporting significant psychological consequences of OASI, including anxiety, shame, fear, and hopelessness [29]. These results highlight a need for improved support for patients after OASI.

Limitations of this study include the timeframe of follow-up. The study was designed to take place after standard postpartum follow-up; therefore, we did not expect such a high proportion of patients to have ongoing symptoms associated with their perineal recovery. Future research could consider assessing recovery at later time points after delivery to evaluate the effect of postpartum interventions in this patient population. Another limitation is that this study did not evaluate economic aspects of care; future research could consider evaluating the cost-effectiveness of telehealth interventions for postpartum pelvic floor recovery. Additionally, while the telehealth consultation included counselling regarding future delivery and discussion of assessments such as endoanal ultrasound when deciding between vaginal versus caesarean for future deliveries, we were unable to evaluate the outcome of this counselling since this study did not include follow-up related to future pregnancies.

The main strength of this study is the prospective design allowing for assessment of the incidence of pelvic floor symptoms at a specific time in recovery without significant recall bias. The pre–post design limits risk of cross contamination between groups whereby individuals counselled in a telehealth consultation would suggest similar interventions to non-telehealth patients via social media groups or mother–baby groups, which are common in our region. The recruitment rate was 89.5% out of all approached patients, and study completion rate of enrolled participants was 93%, suggesting a low risk of non-response bias.

Obstetric anal sphincter injuries have a significant impact on postpartum recovery, with high rates of pain as well as both urinary and flatal/fecal incontinence that persist beyond the standard postpartum visit. A postpartum telehealth consultation focused on pelvic floor health may benefit patients with more severe tears who reported reduced symptom burden as measured by the Pelvic Floor Distress Inventory. Participants rated a telehealth consultation as high value and low burden for this condition.

## Supplementary Information

Below is the link to the electronic supplementary material.

Supplementary file1 (DOCX 69 KB)

Supplementary file2 (DOCX 20 KB)

Supplementary file3 (DOCX 16 KB)

Supplementary file4 (DOCX 17 KB)

Supplementary file5 (DOCX 17 KB)

## Data Availability

The data that support the findings of this study are available from the corresponding author (KB) upon request.
