# Supplementary material for: Evaluating the Benefit of a Urogynecologic Telehealth Consultation after Obstetric Anal Sphincter Injury
Source: Int Urogynecol J. 2025 Jan 31;36(3):677–84. doi: 10.1007/s00192-025-06077-2 (PMC12003585; doi:10.1007/s00192-025-06077-2)
Supplement: Supplementary file 1 — Supplementary file1 (DOCX 69 KB) [file 192_2025_6077_MOESM1_ESM.docx]

# Pelvic Floor Distress Inventory

Instructions: Please answer all of the questions in the following survey. These questions will ask you if you have certain bowel, bladder, or pelvic symptoms and, if you do, how much they bother you. While answering these questions, please consider your symptoms over the **last 3 months**.

The PFDI-20 has 20 items and 3 scales. All items use the following format:

Do you ? If yes, how much does it bother you?

- No ☐ Yes, Not at all ☐ Yes, Somewhat ☐ Yes, Moderately ☐ Yes, Quite a bit

## Do you usually experience *pressure* in the lower abdomen? If yes, how much does it bother you?


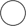
 No


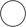
 Yes, Not at all
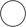
 Yes, Somewhat
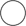
 Yes, Moderately
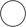
 Yes, Quite a bit

## Do you usually experience *heaviness* or *dullness* in the pelvic area? If yes, how much does it bother you?


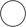
 No


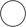

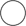
 Yes, Not at all
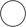
 Yes, Somewhat
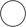
 Yes, Moderately

Yes, Quite a bit

## Do you usually have a bulge or something falling out that you can see or feel in your vaginal area? If yes, how much does it bother you?


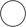
 No


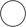
 Yes, Not at all
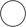
 Yes, Somewhat
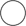
 Yes, Moderately
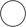
 Yes, Quite a bit

## Do you ever have to push on the vagina or around the rectum to have or complete a bowel movement? If yes, how much does it bother you?


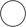
 No


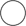
 Yes, Not at all
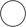
 Yes, Somewhat
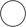
 Yes, Moderately
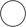
 Yes, Quite a bit

## Do you usually experience a feeling of incomplete bladder emptying? If yes, how much does it bother you?


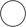
 No


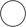

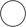
 Yes, Not at all
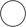
 Yes, Somewhat
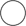
 Yes, Moderately

Yes, Quite a bit

## Do you ever have to push up on a bulge in the vaginal area with your fingers to start or complete urination? If yes, how much does it bother you?


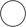
 No


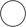
 Yes, Not at all
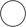
 Yes, Somewhat
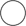
 Yes, Moderately
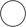
 Yes, Quite a bit

## Do you feel you need to strain too hard to have a bowel movement? If yes, how much does it bother you?


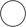
 No


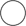
 Yes, Not at all
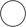
 Yes, Somewhat
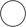
 Yes, Moderately
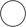
 Yes, Quite a bit

## Do you feel you have not completely emptied your bowels at the end of a bowel movement? If yes, how much does it bother you?


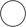
 No


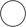

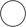
 Yes, Not at all
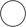
 Yes, Somewhat
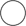
 Yes, Moderately

Yes, Quite a bit

## Do you usually lose stool beyond your control if your stool is well formed? If yes, how much does it bother you?


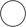
 No


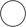
 Yes, Not at all
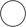
 Yes, Somewhat
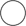
 Yes, Moderately
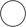
 Yes, Quite a bit

## Do you usually lose stool beyond your control if your stool is loose? If yes, how much does it bother you?


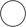
 No


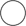
 Yes, Not at all
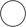
 Yes, Somewhat
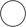
 Yes, Moderately
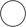
 Yes, Quite a bit

## Do you usually lose gas from the rectum beyond your control? If yes, how much does it bother you?


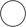
 No


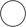

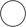
 Yes, Not at all
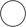
 Yes, Somewhat
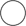
 Yes, Moderately

Yes, Quite a bit

## Do you usually have pain when you pass your stool? If yes, how much does it bother you?


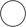
 No


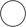
 Yes, Not at all
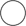
 Yes, Somewhat
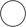
 Yes, Moderately
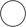
 Yes, Quite a bit

## Do you experience a strong sense of urgency and have to rush to the bathroom to have a bowel movement? If yes, how much does it bother you?


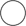
 No


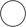
 Yes, Not at all
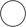
 Yes, Somewhat
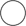
 Yes, Moderately
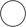
 Yes, Quite a bit

## Does part of your bowel ever pass through the rectum and bulge outside during or after a bowel movement? If yes, how much does it bother you?


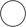
 No


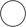

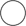
 Yes, Not at all
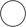
 Yes, Somewhat
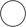
 Yes, Moderately

Yes, Quite a bit

## Do you usually experience frequent urination? If yes, how much does it bother you?


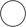
 No


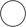
 Yes, Not at all
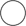
 Yes, Somewhat
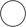
 Yes, Moderately
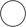
 Yes, Quite a bit

## Do you usually experience urine leakage associated with a feeling or urgency, that is, a strong sensation of needing to go to the bathroom? If yes, how much does it bother you?


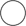
 No


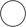
 Yes, Not at all
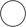
 Yes, Somewhat
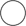
 Yes, Moderately
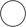
 Yes, Quite a bit

## Do you usually experience urine leakage related to coughing, sneezing, or laughing? If yes, how much does it bother you?


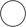
 No


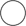
 Yes, Not at all
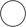
 Yes, Somewhat
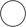
 Yes, Moderately


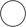
Yes, Quite a bit

## Do you usually experience small amounts of urine leakage (that is, drops)? If yes, how much does it bother you?


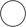
 No


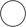
 Yes, Not at all
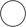
 Yes, Somewhat
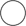
 Yes, Moderately
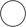
 Yes, Quite a bit

## Do you usually experience difficulty emptying your bladder? If yes, how much does it bother you?


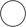
 No


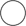
 Yes, Not at all
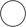
 Yes, Somewhat
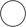
 Yes, Moderately
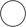
 Yes, Quite a bit

## Do you usually experience pain or discomfort in the lower abdomen or genital region? If yes, how much does it bother you?


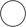
 No


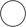

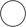
 Yes, Not at all
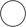
 Yes, Somewhat
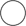
 Yes, Moderately

Yes, Quite a bit

# Female Genital Self-Image Scale

The following items are about how you feel about your own genitals (the vulva and vagina). The word *vulva* refers to a woman’s external genitals (the parts that you can see from the outside such as the clitoris, pubic mound, and vaginal lips). The word *vagina* refers to the inside part, also sometimes called the “birth canal” (this is also the part where a penis may en- ter or where a tampon is inserted). Please indicate how strongly you agree or disagree with each statement.

## Please select the option to indicate how strongly you agree or disagree with each statement.

Strongly

disagree Disagree Agree

Strongly agree

I feel positively about my genitals.

I am satisfied with the appearance of my genitals.

I would feel comfortable letting a sexual partner look at my genitals.

I think my genitals smell fine.

I think my genitals work the way they are supposed to work.

I feel comfortable letting a healthcare provider examine my genitals.

I am not embarrassed about my genitals.

# Female Sexual Function Index

## Over the past 4 weeks:

Very high High Moderate Low

Very low or none at all

How would you rate your level (degree) of sexual desire or interest?

## Over the past 4 weeks:

No sexual

activity Very high High Moderate Low

Very low or none at all

How would you rate your level of sexual arousal (“turn on”) during sexual activity or intercourse?

## Over the past 4 weeks:

No sexual activity

Almost always or

always Most times Sometimes A few times

Almost never or never

How often did you become lubricated (“wet”) during sexual activity or intercourse?

When you had sexual stimulation or intercourse, how often did you reach orgasm?

## Over the past 4 weeks:

Very satisfied

Moderately satisfied

About equally satisfied and not satisfied

Moderately dissatisfied

Very dissatisfied

How satisfied have you been with your overall sexual life?

## Over the past 4 weeks:

Did not attempt intercourse

Almost never or

never A few times Sometimes Most times

Almost always or always

How often did you experience discomfort or pain during vaginal penetration?

# Patient Enablement Instrument

## As a result of your recent medical visits, do you feel you are…

|  | Much better | Better | Same or Less |
| --- | --- | --- | --- |
| Able to cope with life |  |  |  |
| Able to understand your health condition |  |  |  |
| Able to cope with your health condition |  |  |  |
| Able to keep yourself healthy |  |  |  |

1. As a result of your recent medical visits, do you feel you are…

Much more More

Same or Less

Confident about your health

Able to help yourself

# QQ10

## Please select the answer below that best fits your feelings about your prior telehealth consultation.

Strongly agree

Mostly agree

Neither agree or disagree

Mostly disagree

Strongly disagree

It helped me to communicate about my condition.

It was relevant to my condition.

It was straight- forward.

It was too long.

It was too embarrassing

## Please select the answer below that best fits your feelings about your prior telehealth consultation.

The process was too complicated.

It included all the aspects of my condition that I am concerned about.

It upset me.

I would be happy to have a telephone consultation again in the future as part of my routine care.

It was enjoyable
