# Supplementary material for: Evaluating the Benefit of a Urogynecologic Telehealth Consultation after Obstetric Anal Sphincter Injury
Source: Int Urogynecol J. 2025 Jan 31;36(3):677–84. doi: 10.1007/s00192-025-06077-2 (PMC12003585; doi:10.1007/s00192-025-06077-2)
Supplement: Supplementary file 2 — Supplementary file2 (DOCX 20 KB) [file 192_2025_6077_MOESM2_ESM.docx]

Supplemental Table 1: Individual items endorsed on the Pelvic Floor Distress Inventory (PFDI-20), organized by subsection; Pelvic Organ Prolapse Distress Inventory (POPDI-6), Colorectal-Anal Distress Inventory (CRADI-8), and Urinary Distress Inventory (UDI-6).

| Item of PFDI-20 | Control group  (n=62)  Endorsement of item  n (%) | Intervention group (n=57)  Endorsement of item  n (%) | Total  n (%) |
| --- | --- | --- | --- |
| **CRADI-8** |  |  |  |
| Do you feel you need to strain too hard to have a bowel movement? | 29 (46.8%) | 21 (36.8%) | 50 (42.0%) |
| Do you feel you have not completely emptied your bowels at the end of a bowel movement? | 24 (38.7%) | 19 (33.3%) | 43 (36.1%) |
| Do you usually lose stool beyond your control if your stool is well formed? | 7 (11.3%) | 6 (10.5%) | 13 (10.9%) |
| Do you usually lose stool beyond your control if your stool is loose? | 10 (16.1%) | 9 (15.8%) | 19 (16.0%) |
| Do you usually lose gas from the rectum beyond your control? | 29 (46.8%) | 33 (57.9%) | 62 (52.1%) |
| Do you usually have pain when you pass your stool? | 30 (48.4%) | 18 (31.6%) | 48 (40.3%) |
| Do you experience a strong sense of urgency and have to rush to the bathroom to have a bowel movement? | 30 (48.4%) | 29 (50.9%) | 59 (49.6%) |
| Does part of your bowel ever pass through the rectum and bulge outside during or after a bowel movement? | 5 (8.1%) | 7 (12.3%) | 12 (10.1%) |
| **UDI-6** |  |  |  |
| Do you usually experience frequent urination? | 23 (37.1%) | 17 (29.8%) | 40 (33.6%) |
| Do you usually experience urine leakage associated with a feeling or urgency, that is, a strong sensation of needing to go to the bathroom? | 19 (30.6%) | 18 (31.6%) | 37 (31.1%) |
| Do you usually experience urine leakage related to coughing, sneezing, or laughing? | 24 (38.7%) | 21 (36.8%) | 45 (37.8%) |
| Do you usually experience small amounts of urine leakage (that is, drops)? | 27 (43.5%) | 22 (38.6%) | 49 (41.2%) |
| Do you usually experience difficulty emptying your bladder? | 13 (21.0%) | 5 (8.8%) | 18 (15.1%) |
| Do you usually experience pain or discomfort in the lower abdomen or genital region? | 27 (43.5%) | 21 (36.8%) | 48 (40.3%) |
| **POPDI-6** |  |  |  |
| Do you usually experience *pressure* in the lower abdomen? | 19 (30.6%) | 16 (28.1%) | 35 (29.4%) |
| Do you usually experience *heaviness* or *dullness* in the pelvic area? | 24 (38.7%) | 18 (31.6%) | 42 (35.3%) |
| Do you usually have a bulge or something falling out that you can see or feel in your vaginal area? | 10 (16.1%) | 5 (8.8%) | 15 (12.6%) |
| Do you ever have to push on the vagina or around the rectum to have or complete a bowel movement? | 20 (32.3%) | 13 (22.8%) | 33 (27.7%) |
| Do you usually experience a feeling of incomplete bladder emptying? | 21 (33.9%) | 14 (24.6%) | 35 (29.4%) |
| Do you ever have to push up on a bulge in the vaginal area with your fingers to start or complete urination? | 0 | 0 | 0 |
