# Supplementary material for: Evaluating the Benefit of a Urogynecologic Telehealth Consultation after Obstetric Anal Sphincter Injury
Source: Int Urogynecol J. 2025 Jan 31;36(3):677–84. doi: 10.1007/s00192-025-06077-2 (PMC12003585; doi:10.1007/s00192-025-06077-2)
Supplement: Supplementary file 3 — Supplementary file3 (DOCX 16 KB) [file 192_2025_6077_MOESM3_ESM.docx]

Supplemental Table 2: Summary of individual item responses from the Patient Enablement Instrument (PEI). Possible responses for each PEI item were “Much better”, “Better”, or “Same or less” for the first four items, and “Much more”, “More”, or “Same or less” for the last two items.

|  | Control group  (n=61)*  Endorsement of response  n (%) | Intervention group  (n=57)  Endorsement of response  n (%) |
| --- | --- | --- |
| Able to cope with life  “Much better” or “Better” | 35 (57.4%) | 38 (66.7%) |
| Able to understand your health condition  “Much better” or “Better” | 45 (73.8%) | 46 (80.7%) |
| Able to cope with your health condition  “Much better” or “Better” | 43 (70.5%) | 45 (78.9%) |
| Able to keep yourself healthy  “Much better” or “Better” | 37 (60.7%) | 42 (73.7%) |
| Confident about your health  “Much more” or “More” | 37 (60.7%) | 37 (64.9%) |
| Able to help yourself  “Much more” or “More” | 40 (65.6%) | 44 (77.2%) |

*One participant in the control group did not complete the PEI section of the survey.
