# Supplementary material for: Evaluating the Benefit of a Urogynecologic Telehealth Consultation after Obstetric Anal Sphincter Injury
Source: Int Urogynecol J. 2025 Jan 31;36(3):677–84. doi: 10.1007/s00192-025-06077-2 (PMC12003585; doi:10.1007/s00192-025-06077-2)
Supplement: Supplementary file 4 — Supplementary file4 (DOCX 17 KB) [file 192_2025_6077_MOESM4_ESM.docx]

Supplemental Table 3: Summary of individual item responses from the QQ10, organized into “value” items and “burden” items (Moores et al., 2012).

Note that the QQ10 was only completed by the intervention group (n=57), since its purpose was to evaluate the telehealth consultation.

|  | “Mostly agree” *or* “Strongly agree”  n (%) | “Neither agree or disagree”  n (%) | “Mostly disagree” *or* “Strongly disagree”  n (%) |
| --- | --- | --- | --- |
| **“Value” items** |  |  |  |
| It helped me to communicate about my condition. | 46 (80.7%) | 10 (17.5%) | 1 (1.8%) |
| It was relevant to my condition. | 51 (89.5%) | 5 (8.8%) | 1 (1.8%) |
| It was straight-forward. | 53 (93.0%) | 3 (5.3%) | 1 (1.8%) |
| It included all the aspects of my condition that I am concerned about. | 45 (78.9%) | 6 (10.5%) | 6 (10.5%) |
| I would be happy to have a telephone consultation again in the future as part of my routine care. | 42 (73.7%) | 11 (19.3%) | 4 (7.0%) |
| It was enjoyable. * | 29 (51.8%) | 25 (44.6%) | 2 (3.6%) |
| **“Burden” items** |  |  |  |
| It was too long. | 4 (7.0%) | 15 (26.3%) | 38 (66.7%) |
| It was too embarrassing | 3 (5.3%) | 12 (21.1%) | 42 (73.7%) |
| The process was too complicated. | 2 (3.5%) | 9 (15.8%) | 46 (80.7%) |
| It upset me. | 1 (1.8%) | 8 (14.0%) | 48 (84.2%) |

*1 participant did not answer this question.
