# Supplementary material for: Evaluating the Benefit of a Urogynecologic Telehealth Consultation after Obstetric Anal Sphincter Injury
Source: Int Urogynecol J. 2025 Jan 31;36(3):677–84. doi: 10.1007/s00192-025-06077-2 (PMC12003585; doi:10.1007/s00192-025-06077-2)
Supplement: Supplementary file 5 — Supplementary file5 (DOCX 17 KB) [file 192_2025_6077_MOESM5_ESM.docx]

Supplemental Table 4: Summary of data from telehealth consultations, n=57 (intervention group).

| **Characteristic** | **Number (%)** |
| --- | --- |
| Perineal pain | 29 (50.9%) |
| Urinary incontinence | 15 (26.3%) |
| Flatal incontinence | 21 (36.8%) |
| Fecal incontinence | 10 (17.5%) |
| Pelvic organ prolapse symptoms | 0 |
| Engaged with pelvic floor physiotherapy since delivery | 30 (52.6%) |
| Use of vaginal estrogen postpartum | 7 (12.3%) |
| Referral made during telehealth consult | 24 (42.1%) |
| - Pelvic floor physiotherapy | 19 (33.3%) |
| - Urogynecology | 3 (5.3%) |
| - Gynecology | 1 (1.8%) |
| - Endoanal ultrasound | 1 (1.8%) |
| Prescription provided for vaginal estrogen during telehealth consult | 23 (40.4%) |
